# Limited resilience of the soil microbiome to mechanical compaction within four growing seasons of agricultural management

**DOI:** 10.1038/s43705-021-00046-8

**Published:** 2021-08-31

**Authors:** Manon Longepierre, Franco Widmer, Thomas Keller, Peter Weisskopf, Tino Colombi, Johan Six, Martin Hartmann

**Affiliations:** 1 Sustainable Agroecosystems, Department of Environmental Systems Science, ETH Zurich, Zurich, Switzerland; 2 Molecular Ecology, Agroscope, Zurich, Switzerland; 3 Soil Quality and Soil Use, Agroscope, Zurich, Switzerland; 4 Department of Soil and Environment, Swedish University of Agricultural Sciences (SLU), Uppsala, Sweden

**Keywords:** Soil microbiology, Biodiversity, Molecular ecology, Microbial ecology

## Abstract

Soil compaction affects many soil functions, but we have little information on the resistance and resilience of soil microorganisms to this disturbance. Here, we present data on the response of soil microbial diversity to a single compaction event and its temporal evolution under different agricultural management systems during four growing seasons. Crop yield was reduced (up to −90%) in the first two seasons after compaction, but mostly recovered in subsequent seasons. Soil compaction increased soil bulk density (+15%), and decreased air permeability (−94%) and gas diffusion (−59%), and those properties did not fully recover within four growing seasons. Soil compaction induced cropping system-dependent shifts in microbial community structures with little resilience over the four growing seasons. Microbial taxa sensitive to soil compaction were detected in all major phyla. Overall, anaerobic prokaryotes and saprotrophic fungi increased in compacted soils, whereas aerobic prokaryotes and plant-associated fungi were mostly negatively affected. Most measured properties showed large spatial variability across the replicated blocks, demonstrating the dependence of compaction effects on initial conditions. This study demonstrates that soil compaction is a disturbance that can have long-lasting effects on soil properties and soil microorganisms, but those effects are not necessarily aligned with changes in crop yield.

## Introduction

Modern agriculture depends on a high level of mechanization to efficiently and economically manage cropping systems. However, the use of heavy machinery often leads to soil compaction, in particular during field operations under unfavorable soil conditions, such as wet soils [1]. Once soil is compacted, it can take decades for the soil to recover [2] without appropriate management [3]. The European commission and the FAO recognize soil compaction as a major threat to soils [4], and emphasize the need to assess the severity of compaction and its effect on ecosystem functioning, in order to develop appropriate regulations [5].

Soil compaction adversely affects soil structure by increasing bulk density and soil mechanical resistance, as well as reducing macroporosity and pore connectivity [6, 7]. As a result, water and gas transport capacities of soil are reduced, leading to poor water infiltration and drainage, as well as low soil aeration [8]. Consequently, soil compaction also affects soil chemical reactions that depend on oxygen concentration and water availability [9, 10]. Compaction is often most severe in the topsoil [9], but most persistent in the subsoil [2, 11]. The largest impact of compaction on soil organisms likely occur in the topsoil, since this is where most of the root biomass [12] and microbial biomass and diversity [13] are contained, but also represents the preferred habitat for most of the soil fauna, such as earthworms [14] and collembola [15]. Therefore, topsoil is most likely the most sensitive layer to measure both the initial impact and first signs of resilience after a compaction event.

Compaction affects soil biological processes in multiple ways, and effects could be direct, e.g., increased mechanical resistance for bioturbation, or indirect via changes of the soil environment, e.g., soil oxygen and moisture levels. Soil mechanical resistance increases under compaction, and can lead to a decrease in root elongation and rooting depth [16], resulting in a reduced accessibility to water and nutrients. As a consequence, soil compaction can cause substantial reductions in crop yield [17].

Pore space accessibility for bacteria and fungi can become limited in compacted soils [18], resulting in a reduction of soil microbial biomass [19]. Moreover, soil compaction can impact soil microbial metabolism by largely limiting aerobic processes, such as nitrification and mineralization [20, 21]. Indeed, the decrease in macropores [22] and oxygen diffusion [23] can lead to anoxic conditions in compacted soils, if oxygen consumption is faster than its supply [24]. As a consequence, obligate and facultative anaerobes might have physiological advantages in compacted soils [20]. Changes in microbial community composition and activity under compaction can shift carbon and nitrogen metabolism toward reduced soil basal respiration, and increased methanogenesis and denitrification [19]. As a result, carbon dioxide emissions are reduced, and methane and nitrous oxide emissions increased [25].

There is a relatively good understanding of soil compaction impact on physical soil properties, but we still lack comparable knowledge about its consequences for the soil microbiome. Most studies have focused on microbial biomass and functional activity of certain microbial processes, for example, greenhouse gas fluxes [20, 26]. Methodological constraints have, however, long limited our ability to characterize soil microbial diversity. Changes in microbial diversity due to compaction have previously been assessed using techniques, such as phospholipid fatty acids or terminal restriction fragment length polymorphism analyses that provide compositional information at a relatively coarse level of resolution, and without much power for taxonomic identification of the responsive groups [27, 28]. Recent studies have harnessed the potential of high-throughput DNA sequencing technologies to assess the resilience of soil microbial communities to soil compaction in forest ecosystems [25, 29]. However, the results from forests cannot be directly translated to agricultural systems as these two ecosystems differ fundamentally in their management. As a consequence, high-throughput DNA sequencing assessment of soil compaction effects on soil microbiome in various arable fields is missing but needed.

The aim of this study was to assess the impact of soil compaction on soil microbial diversity, and its temporal evolution under different agricultural management systems in the first four growing seasons following compaction. Such information is essential to link our knowledge of changes in soil physicochemical properties and crop yield [11, 30] to the ecosystem functions mediated by microorganisms in arable fields. For this purpose, we sampled a long-term field experiment [11] where a single compaction event was implemented in 2014 and subsequently four recovery treatments were established, i.e., permanent ley (PL), bare soil (BS), crop rotation under conventional tillage (CT), and crop rotation under no tillage (NT).

Based on previous observations in forest ecosystems [25], we hypothesized that soil compaction alters soil microbial community structures by promoting anaerobically respiring prokaryotes and saprobic fungi, as well as limiting aerobically respiring prokaryotes and plant-associated fungi. We expected that one single compaction event would have an impact in the short term, but that the microbial community would largely recover over four growing seasons. We further hypothesized that the crop rotations, in particular those including tillage operations, would show faster recovery rates than the permanent ley.

## Materials and methods

### Experimental design

**Fig. 1:**
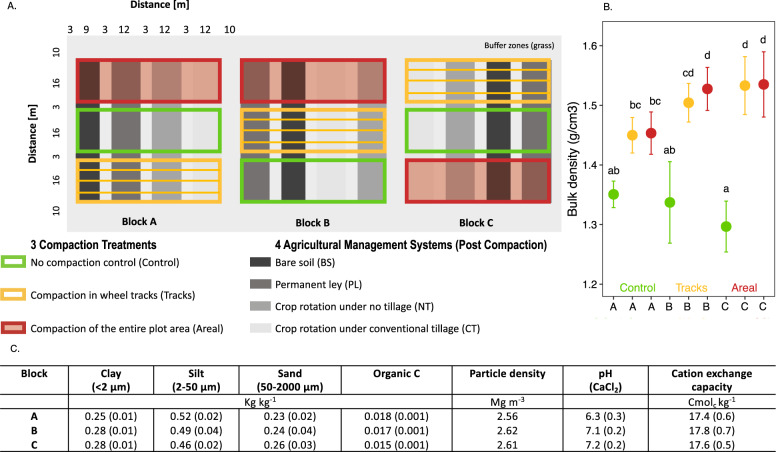
Description of the Soil Structure Obvervatory (SSO). **A** The experimental design of the long-term SSO modified from Keller et al. [11]. The design includes two different compaction treatments and an uncompacted control examined under four different post-compaction management systems, replicated in three blocks of 54 m × 54 m. **B** Differences in bulk densities across the three different blocks (A, B, and C) and across the three compaction treatments (“control”, “tracks”, and “areal”). The data represents the mean (±s.d.) from each condition with *n* = 9. Different letters indicate significant differences between each block and treatment as obtained by Dunn’s test. **C** Basic physicochemical properties of the topsoil prior to compaction showing mean (±s.d.) (modified from Keller et al. [11]).

The Soil Structure Observatory (SSO, Fig. 1A) was established in 2014 at the Agroscope research station in Zurich, Switzerland (47.4° N, 8.5° E; 444 m asl) [11]. In preparation for the compaction experiment, the site was sown with a ley mixture (grass–legume) in spring 2013. Two different soil compaction treatments, i.e., compaction in wheel tracks (hereafter called “tracks”) and compaction of the entire plot area (“areal”), were inflicted with a two-axle self-propelled agricultural vehicle (wheel load 8 Mg, 1050/50R32 tires, inflation pressure 300 kPa) in April 2014, and compared to a non-compacted control treatment (“control”). The “tracks” compaction treatment included three passages of the vehicle leading to six-wheel tracks per plot. The track width was equal to 1 m, the distance between left and right tire track was 1 m, and the distance between adjacent vehicle passages was 2 m. After the compaction event, four different post-compaction agricultural management systems were established, including PL, BS, NT, and CT. Each combination (three compaction treatments × four agricultural management systems) was replicated three times in a strip-plot design, i.e., three field blocks, with each of the 36 plots being 16 m × 12 m in size except for BS that had a plot size of 16 m × 9 m (Fig. 1A). In this study, we report recovery process after compaction only for PL, NT, and CT. Basic physicochemical soil properties, such as soil texture, organic carbon, and pH slightly varied across the blocks prior to compaction (Fig. 1C). Moreover, the soil is characterized as a pseudogley with temporary waterlogging due to a shallow water table that varies between blocks (Thomas Keller, personal communication). The combination of the initial differences in multiple physicochemical soil properties, as well as a potential variation in soil moisture levels may have led to different degrees of compaction across the three blocks, as indicated by the measured bulk densities (Fig. 1B).

No machinery traffic or tillage was permitted in PL. The ley was cut four to five times per year using a one-axle self-propelled hand mover, and the harvested ley was manually removed from the plots. In the crop rotations, some traffic was allowed for fertilizing, spraying, seeding, and harvesting; however, the used equipment had much smaller tire load than the machine used for the compaction event and any additional impact would be consistent across all compaction treatments including the uncompacted “control”. Fertilization was performed according to the Swiss fertilization recommendation (GRUDAF) [31] and crop protection (weed and disease control) according to the principles of “integrated pest management”. Seeding was done with a no-till drill in the crop rotation without tillage plots, while the soil was moldboard plowed to ~0.25 m and harrowed to about the 0.06-m depth using a rotavator in the crop rotation with tillage plots. The tillage practices were done 8 days after the soil compaction event in April 2014 and therefore before any sampling campaign.

### Permanent ley and arable crops

The PL (grass–legume) mixture is based on the mixture SM442 [32] constituted of ryegrass (*Lolium perenne*; 3 kg ha^−1^), Kentucky bluegrass (*Poa pratensis*; 10 kg ha^−1^), Timothy (*Phleum pretense*; 3 kg ha^−1^), creeping red fescue (*Festuca rubra*; 4 kg ha^−1^), tall fescue (*Festuca arundinacea*; 8 kg ha^−1^), and white clover (*Trifolium repens* L*. and Trifolium repens* H*.*; 5 kg ha^−1^), supplemented with 4 kg ha^−1^ of Lucerne (*Medicago sativa* L*.*). For harvesting, two frames (0.5 m × 0.5 m each) were placed per plot and the grass was cut within each frame, and weighed (dry biomass) in 2014 and 2017. The crop rotation consisted of triticale (*X Triticosecale*, 2014), silage maize (*Zea mays* L., 2015), winter wheat (*Triticum aestivum* L., 2016), and winter rapeseed (*Brassica napus* L., 2017). Crop yield was measured as grain weight for triticale, wheat, and rapeseed, and as dry above-ground biomass for silage maize for each growing season.

### Soil physical properties

Soil samples for physical properties were collected in fall 2013 ~6 months before the compaction, as well as a couple of weeks after compaction (spring 2014), 1 year (spring 2015), 2 years (spring 2016), and 3 years (spring 2017) after the compaction event. At each time point, three undisturbed cylindrical soil cores (100 cm^3^; diameter: 0.05 m; height: 0.05 m) per field block, compaction treatment, and agricultural management system were sampled from the topsoil. Bulk soil cores were collected at fixed distances around three random GPS coordinates within each field block for both “control” and “areal” compaction treatment, and inside the wheel tracks for the “tracks” compaction treatment. Sampling in close vicinity to the plants was avoided. Soil cores were analyzed for bulk density, gas diffusion and air permeability directly after compaction (spring 2014) and 3 years after compaction (spring 2017). Air permeability was obtained by measuring the air flow through the sample at an overpressure of 2 hPa, and gas diffusivity was measured in a one-chamber apparatus that uses O_2_ as the diffusing gas assuming steady-state diffusion [11]. The soil cores were dried in an oven at 105 °C for at least 48 h after the various measurements, and bulk density were calculated, as described previously [11].

### Microbial community structure and gene abundance

Two sampling events were done prior to compaction, one in fall 2013 ca. 6 months before compaction and another one a few weeks before compaction (spring 2014). In total, 104 randomized samples (52 × 2) were collected at evenly spaced locations across the three blocks to assess initial heterogeneity of the field. After the compaction event, soil samples were collected after half a year (fall 2014), 1 year (spring 2015), 2 years (spring 2016), and 3 years (spring 2017). At each sampling event, bulk soil samples (0–20 cm) were collected by pooling four soil cores (2 cm diameter) located at three randomly selected positions within each field block for both “control” and “areal” compaction treatment, and inside the wheel tracks for “tracks” compaction treatment, alongside the physical property sampling spots, giving a total of 81 soil samples per time point (three field blocks × three replicates per block × three compaction treatments × three agricultural management systems). Soils were transported on ice to the laboratory, immediately sieved (mesh size of 4 mm), and stored at −20 °C until nucleic acid extraction.

Nucleic acids were extracted from 0.5 g soil according to the protocol of Bürgmann et al. [33] using a bead-beating procedure on the FastPrep-24 5 G (MP Biomedicals, Irvine, CA, USA). Extracted DNA was further purified using the NucleoSpin gDNA Clean‑up kit (Macherey Nagel, Düren, Germany), examined with gel electrophoresis, and quantified using a PicoGreen^®^ assay (Molecular Probes, Eugene, OR, USA). DNA concentrations were adjusted to 5 ng μl^−1^ in H_2_O containing bovine serum albumin (BSA, 10 v/v% of 10 mg ml^−1^) and heated to 95 °C to bind potential PCR-inhibiting substances, such as humic acids.

For sequencing, PCR amplification of the bacterial and archaeal (V3 and V4 regions of the 16S rRNA gene)—further termed prokaryotic—as well as fungal (ITS2 region of the rrn operon) markers was performed as described previously [34], using primers 341F and 806R for bacteria and archaea [34] and ITS3ngs and ITS4ngs for fungi [35] on 40 ng DNA. PCR amplification was carried out in technical triplicates and products were pooled prior to sequencing. PCR products were sent to the Génome Québec Innovation Center (Montréal, Canada) for barcoding using the Fluidigm Access Array technology (Fluidigm, South San Francisco, CA, USA) and paired-end sequencing on the Illumina MiSeq v3 platform (Illumina Inc., San Diego, CA, USA).

For functional gene quantification via quantitative PCR (qPCR), the lack of amplification inhibition was tested by qPCR amplification of the pGEM-T plasmid (GenBank® Accession No. X65308, Promega, Madison, WI, USA) with the plasmid specific primers SP6 and T7, introduced at equimolar concentration in all samples, in order to assess the amplification variability across treatments. The DNA standards were prepared from purified PCR products obtained by amplifying the targets from a pool of DNA from all samples. The concentrations used for the standard curves ranged from 10^−2^ to 10^−7^ ng of DNA per reaction. qPCR was performed on 10 ng DNA with specific primers targeting the 16S rRNA gene (515F/806R) [36], the *nir*K gene (5F/890F) [37], and the *nir*S gene (CD3aF Throback/ R3cd Throback) [38]. All the reactions were performed with a concentration of 1 µM of each primer, 1× SSO Advanced^TM^ Universal SYBR® Green Supermix (Bio-Rad Laboratories, Hercules, CA, USA), and 0.6 mg ml^−1^ of BSA. The conditions for each qPCR were as follows: 3 min for enzyme activation at 98 °C, then a cycle of 15 s for denaturation at 95 °C, 30 s primer hybridization at 58 °C for *nir*K and *nir*S or at 52 °C for 16S, and 30 s for extension at 72 °C was carried out 30 times for *nir*K and *nir*S primers and 35 times for 16 S primers. Prior to the previous detailed qPCR cycles, six cycles of touch down qPCR for *nir*K and *nir*S primer were done with hybridization at 63–1 °C/cycle for 30 s (the other steps being unchanged). Melting curves were generated by increasing the temperature from 75 to 95 °C by 0.5 °C every 5 s at the end of the amplification cycles, in order to verify the amplification specificity. The qPCR efficiencies (E) were equal to 97.4 (*R*^2^ = 0.999) for the 16S rRNA gene, 99.5 (*R*^2^ = 0.998) for the *nir*K gene, and 94.8 (*R*^2^ = 0.995) for the *nir*S gene. Estimated copy numbers for each targeted gene were calculated by the following formula:

$${{Number}}\;{{of}}\;{{copies}}\;{{per}}\; \mu {{L}} = \frac{{6.022 \times 10^{23}\left( {{molecules}}/{{mole}} \right) \times {{DNA}}\;{{concentrations}}\;({{g}}/\mu{{L}})}}{{{Number}}\;{{of}}\;{{base}}\;{{pairs}}\;{{of}}\;{{the}\;{{targeted}\;{{gene}}\,\times 660\;{{(daltons)}}}}}$$

6.022 × 10^23^ being the Avogadro’s number; 660 being the average weight of a single base pair. The length of each target was estimated by in silico PCR with the primers used on all gene-specific sequences obtained from the NCBI nucleotide database and averaging the amplicon size.

### Bioinformatic analysis

The microbial sequence data were processed using a customized pipeline largely based on VSEARCH [39]. PCR primers were trimmed using CUTADAPT [40] allowing for one mismatch and filtered for PhiX contamination by running the reads against the PhiX genome (accession NC_001422.1) using Bowtie2 [41]. Trimmed paired-end reads were merged using the *fastq_mergepairs* function and quality filtered using the *fastq_filter* function with a maximum expected error of one [42] both implemented in VSEARCH. Sequences were dereplicated using the *derep_fulllength* function in VSEARCH and delineated into amplicon sequence variants (ASVs) using the UNOISE algorithm [43] in VSEARCH, with an alpha of 2 and a minsize of 4. Potentially chimeric ASV sequences were identified and removed using the UCHIME2 algorithm [44] implemented as the *uchime3_denovo* function in VSEARCH. Remaining ASV sequences were tested for the presence of ribosomal signatures, using Metaxa2 [45] and ITSx [46] for the 16S rRNA gene and ITS2 sequences, respectively, and unsupported sequences were discarded. The final ASV table was obtained by mapping the quality filtered reads of each sample against the verified ASV sequences using the *usearch_global algorithm* implemented in VSEARCH with settings maxrejects 100, maxaccepts 0, maxhits 1, and a minimum identity of 97%. Taxonomic classification of each verified ASV sequence was performed by running the SINTAX algorithm [47] implemented in VSEARCH against the SILVA v.132 database [48] for the 16S rRNA gene sequences (bacteria and archaea) and against the UNITE v.7.2 database [49] for the ITS2 sequences (fungi), using a bootstrap cutoff of 0.8. ASVs not assigned at the domain level of bacteria, archaea, or fungi, as well as ASVs assigned to organelle structures (chloroplasts and mitochondria) were removed from the ASV table. The average number of sequences for the prokaryotic and fungal datasets were 19,020 (±4700) and 20,225 (±6005), respectively. Raw sequences were deposited in the European Nucleotide Archive under the accession number PRJEB43264.

### Statistical analysis

All statistical analyses were performed in R [50] and *p* values < 0.05 were considered significant in all statistical tests. Change in yields (kg ha^−1^) for PL and arable crops in the rotations were presented as percent change in comparison to (a) PL in the uncompacted control or (b) the respective crop in the uncompacted control under CT, respectively. For PL, the significant differences in yield between the control and the compaction treatments were assessed using Kruskal–Wallis test with the *kruskal.test* function in R, since the homogeneity of variance and normal distribution of the residuals were not supported. For the arable crop in the rotation, the significant differences in yield over all blocks between the control and the compaction treatments, as well as the tillage effect within each year were assessed with ANOVA using the *aov* function in R since the homogeneity of variance and normal distribution of the residuals were supported. The multiple pairwise comparisons were done by the Tukey test with the *TukeyHSD* function in R from the package *stats*.

Significant differences of the soil physical properties (e.g., bulk density, air permeability, and gas diffusion) between the various conditions were assessed using the Kruskal–Wallis test with the *kruskal.test*, as described earlier. The multiple pairwise comparisons were done by Dunn’s test with the *dunn.test* function in R from the package dunn.test v.1.3.5 [51].

Sequencing depth was examined using barplots and rarefaction curves with the *rarecurve* function from the *vegan* package [52]. In order to account for differences in sequencing depth (Supplementary Fig. 1), differences in α-diversity (observed richness, Pielou’s evenness, and Shannon diversity) and β-diversity (Bray–Curtis dissimilarity) were determined from ten iteratively subsampled and square-root transformed ASV count tables [53, 54] using the *rrarefy*, *specnumber*, *diversity*, and *vegdist* functions in *vegan*. The effect of space (block), time (date), compaction treatment, and agricultural management systems on α-diversity and on β-diversity were assessed using univariate or multivariate permutational analysis of variance (PERMANOVA [55]) as implemented in the *adonis* function from *vegan* using 999 permutations. Pairwise tests between factor levels were performed using the *pairwise.perm.manova* function implemented in package *RVAideMemoire* v.0.9–73 [56]. Differences in β-diversity were assessed by unconstrained ordination using principal component analysis [57] with the *cmdscale* function in R and by constrained ordination using canonical analysis of principal coordinates (CAP [58]) with the *CAPdiscrim* function of the *BiodiversityR* package [59], with 999 permutations using the factors labeled as significant in the PERMANOVA as constraining factor. Temporal response of individual taxa to soil compaction (i.e., initial sensitivity and resilience over time) was assessed, using PERMANOVA via the *adonis* function with 999 permutations with spatial partitioning to account for the spatial effect. Adjustments for multiple testing were performed using *q* values [60] implemented in the *qvalue* function of the R package *qvalue* v.2.16.0 [61] and *q* values < 0.05 were considered significant. To avoid inflation of type II error due to the impact of rare and infrequent taxa on multiple testing correction, ASVs with an overall abundance <0.5% and/or occurring in less than four samples were not included in the test, removing ~90% of the ASV for prokaryotes and ~77% for fungi. The taxonomic trees for the prokaryotic and fungal ASVs assigned at the genus level showing a significant increase or decrease in their relative abundance under “tracks” or “areal” compaction treatments, when compared to “control” within each agricultural management systems were generated with iToL v6.1.2 [62] based on a tree matrix retrieved form the taxonomy table using the *taxa2dist* function from the *vegan* package, and the *hclust* function from the *ade4* package, respectively. In order to make inferences with respect to the potential lifestyle of the taxa, literature searches were performed supported by the literature also available through Faprotax v1.2.4 [63] and FUNGuild v.1.0 [64] for prokaryotes and fungi, respectively.

The estimated copy numbers per gram of dry soil of the *nir*S and *nir*K genes were normalized by dividing the values by the 16S rRNA gene copies, and significant differences in relative estimated copy numbers between the various conditions were assessed using ANOVA with the *aov* function, as described earlier.

## Results

### Impact of compaction on soil physical properties

**Fig. 2:**
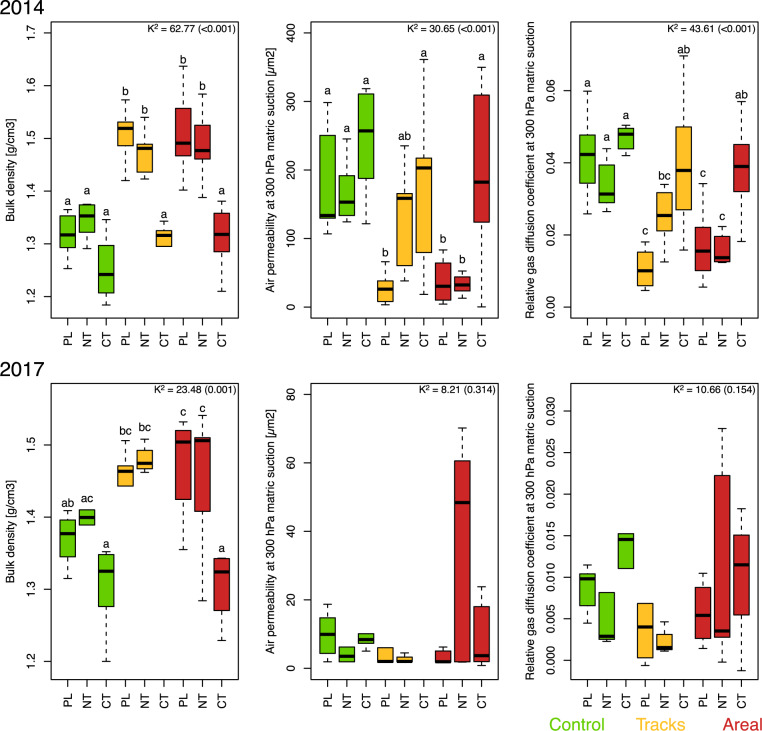
Agricultural management-dependent effects of soil compaction on soil physical properties. Soil bulk density, air permeability at 300 hPa matric suction, and relative gas diffusion coefficient at 300 hPa matric suction in the “control”, “tracks”, and “areal” compaction treatments for the permanent ley (PL), as well as the crop rotations with conventional tillage (CT) and no tillage (NT) in 2014 and 2017 (soil physical data for “tracks” under CT in 2017 are missing). Different letters indicate significant differences within each year, between compaction treatments and agricultural management systems as obtained by Dunn’s test.

In PL and NT, soil compaction significantly increased soil bulk density by ~10–15%, and significantly reduced air permeability by 60–94% and gas diffusion by 48–66% (Fig. 2). In the fourth growing season after compaction, soil bulk density had not recovered much in the two agricultural management systems without mechanical loosening, i.e., PL and NT, while gas diffusivity and air permeability had improved; however, some differences remained but were not statistically supported again due to the variation between blocks (Fig. 2). In contrast, the measured soil physical properties mostly recovered under CT after mechanical loosening in spring 2014 (Fig. 2).

### Permanent ley biomass and arable crop yield

**Fig. 3:**
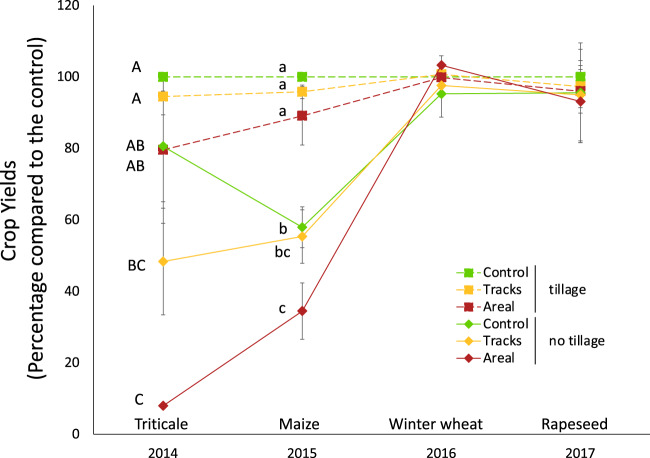
Agricultural management-dependent effects of soil compaction on crop yields. Yearly differences in crop yields under “tracks” and “areal” compaction treatment in the crop rotations under the conventional and no-tillage systems from 2014 to 2017 expressed as percentage in comparison to the yield in the uncompacted tillage system as a reference. The data represents the mean (±s.e.) from each condition with *n* = 3. Different letters indicate significant differences within each year (capital letters for 2014 and small letters for 2015) as obtained by Tukey’s HSD.

Ley biomass in compacted plots was ~20% lower in 2014 (81 ± 21% of the biomass in the uncompacted control) and 30% lower in 2017 (68 ± 33%) than in “control” plots, although the differences were not statistically significant due to the large variation between blocks. In the crop rotations, compared to the yield in the uncompacted tillage treatment, the average yield of triticale (2014) and maize (2015) in NT was ~51–58% under the “tracks” compaction (affecting about one third of the total plot area) and ~8–35% under the “areal” compaction (Fig. 3). Both differences between “tracks” and the uncompacted tillage treatment were, however, not statistically significant due to the large variation between blocks (Fig. 3). Yields of winter wheat (2016) and winter rapeseed (2017) were not or only marginally affected by both soil compaction treatments. Under CT, soil compaction resulted in at least 79% (triticale, 2014) and 90% (maize, 2015) of the yield of the uncompacted tillage treatment, but those reductions compared to the uncompacted tillage treatment were not statistically significant. Only marginal reductions occurred for winter wheat (2016) and winter rapeseed (2017; Fig. 3).

### Soil microbial diversity

Observed richness, Pielou’s evenness, and Shannon diversity of the prokaryotic community did not show any differences due to soil compaction (e.g., tracks or areal) and between the different agricultural management systems (e.g. PL, NT, and CT; Supplementary Fig. 2 and Supplementary Table 1). In contrast, soil fungal richness showed a significant increase under “areal” compaction when compared to “control” and “tracks”, but the Pielou’s evenness and Shannon diversity were not significant between conditions. Moreover, the observed richness, Pielou’s evenness and Shannon diversity of fungal community also showed a significant decrease in NT, when compared to PL and CT (Supplementary Fig. 3 and Supplementary Table 1). Significant differences in prokaryotic richness, evenness, and Shannon diversity were observed across the different years and the different blocks, with lower values in the first two growing seasons (2014, 2015), when compared to the last two investigated seasons (2016, 2017), as well as lower values in block A than in blocks B and C (Supplementary Fig. 2 and Supplementary Table 1). Fungal richness tended to be significantly higher in 2015 and 2016 when compared to 2014 and 2017 (Supplementary Fig. 3 and Supplementary Table 1), as well as significantly higher in block A when compared to blocks B and C. Soil fungal evenness and Shannon diversity did not show any differences across blocks and time (Supplementary Fig. 3 and Supplementary Table 1).

**Fig. 4:**
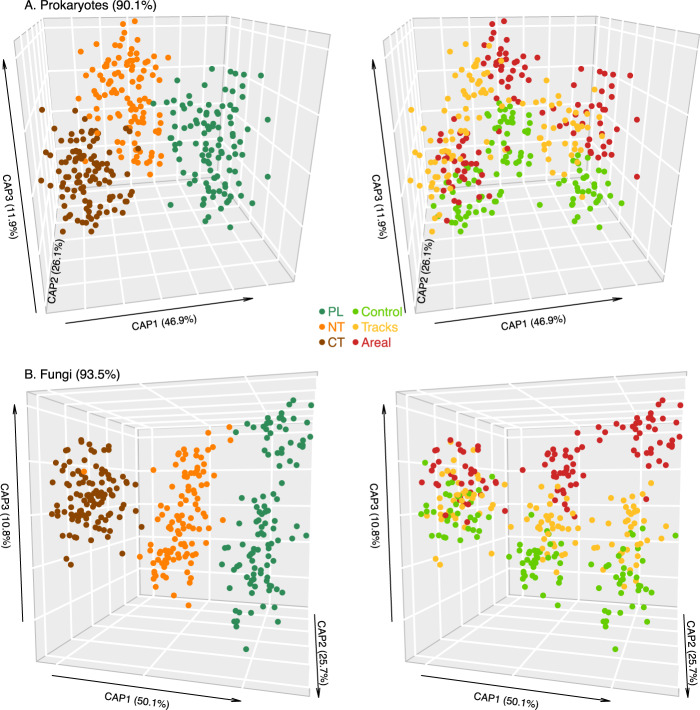
Agricultural management-dependent effects of soil compaction on microbial community structure. Differences in soil prokaryotic (**A**) and fungal (**B**) community structure across the three different agricultural management systems, including the permanent ley (PL, green), as well as the crop rotations with conventional (CT, brown) and no tillage (NT, orange) in response to the full areal (red) and track only (yellow) compaction treatments in comparison with the uncompacted control (light green), as obtained by canonical analysis of principal coordinates (CAP). The amount of between group variation of each CAP axis is provided in parenthesis. The overall CAP reclassification success rates for both CAP models are provided in parentheses next to the domain name. The CAP reclassification success rate provides a quantitative estimation of the degree of discrimination between treatment groups.

Stronger shifts were observed for prokaryotic and fungal β-diversity (Table 1 and Fig. 4). The agricultural management system showed stronger effects on fungi (7%) than on prokaryotes (2%), whereas soil compaction explained ~1 and 2% of the variance for prokaryotes and fungi, respectively (Table 1). Each compaction treatment and agricultural management system harbored a statistically (*p* < 0.045) distinct microbial community over the whole experiment (Fig. 4). Soil compaction effects on microbial community structures depended on the agricultural management system (Table 1 and Fig. 4). Systems with PL (Fig. 4, green) showed distinct microbial communities under “tracks” and “areal” compaction when compared to “control”, as also demonstrated by the high CAP reclassification success rates of 92–97% and 89–100% for prokaryotes and fungi, respectively (Fig. 4). The CAP reclassification success rate provides a quantitative estimation of the degree of discrimination between treatment groups. NT (Fig. 4, orange) showed some differences between the compacted and control plots with high CAP reclassification success rates of 89–92% and 94–97% for prokaryotes and fungi, respectively. CT (Fig. 4, brown) featured the smallest differences between the treatments with CAP reclassification success rates of 72–86% and 88–97% for prokaryotes and fungi, respectively. The differences in prokaryotic and fungal community structure between compacted (“tracks” and “areal”) and non-compacted soils did not change significantly over time for any of the three management systems, since there were no significant interactions between compaction treatment and time, as well as between compaction treatment, agricultural management system, and time (Table 1).

**Table 1:** Soil compaction and agricultural management effects on soil microbial β-diversity.

	Bacteria			Fungi		
	PERMANOVA^a^		PERMDISP^b^	PERMANOVA^a^		PERMDISP^b^
	*F* (*P*)	*R*^2^	*F* (*P*)	*F* (*P*)	*R*^2^	*F* (*P*)
Treatment	1.91 (0.003)	0.01	0.58 (0.531)	3.74 (0.001)	0.02	1.11 (0.325)
Management	2.89 (0.001)	0.02	1.62 (0.201)	16.47 (0.001)	0.07	0.27 (0.758)
Date	3.29 (0.001)	0.03	0.20 (0.885)	12.74 (0.001)	0.08	84.41 (0.001)
Block	33.49 (0.001)	0.17	25.19 (0.001)	34.62 (0.001)	0.15	3.35 (0.037)
Treatment × management	1.29 (0.039)	0.01	0.60 (0.767)	1.94 (0.001)	0.02	21.4 (0.001)
Treatment × date	0.96 (0.590)	0.01	0.23 (0.994)	0.96 (0.606)	0.01	0.24 (0.985)
Management × date	1.09 (0.199)	0.02	0.44 (0.934)	3.26 (0.001)	0.04	12.13 (0.001)
Treatment × management × date	0.93 (0.764)	0.03	0.18 (1)	0.93 (0.819)	0.02	2.59 (0.001)
Residual		0.71			0.60	

^a^Effects of main factors and their interactions as assessed by multivariate permutational analysis of variance (PERMANOVA). Values indicate the *F*-ratio (*F*), the level of significance (*P*), and the explained variance (*R*^2^).

^b^Heterogeneity of variance assessed by permutational analysis of multivariate dispersion (PERMDISP). Values indicate the *F*-ratio (*F*) and the level of significance (*P*).

The strongest shifts for prokaryotic and fungal β-diversity were due to the spatial and temporal variability. The spatial effect was the dominating factor (15–17%) shaping the microbial community structure with each field block having significantly (*p* < 0.001) distinct microbial communities (Table 1 and Supplementary Fig. 4C, D). However, based on the pre-compaction data from year 2013, this β-diversity gradient through the experimental field was already present before establishing the experiment (Supplementary Fig. 4A, B). Another major significant (*p* < 0.029) shift in microbial community structure was observed over time (3–8%; Table 1), in particular from 2015 to 2016 (Supplementary Fig. 4E, F).

### Compaction-sensitive microbial taxa

After correcting for multiple testing, ~24% (6% assigned at genus level) out of the 3871 prokaryotic ASVs and ~43% (13% assigned at genus level) out of the 1141 fungal ASVs responded significantly to soil compaction, i.e., “tracks” versus “control” and/or “areal” versus “control”, across the different agricultural management systems. Most of these sensitive ASVs (increasing or decreasing under compaction) were unique for a specific agricultural management system and only very few ASVs were responding across two (up to 31) or all three management systems (up to 10) under “tracks” or “areal” compaction (Supplementary Fig. 5).

**Fig. 5:**
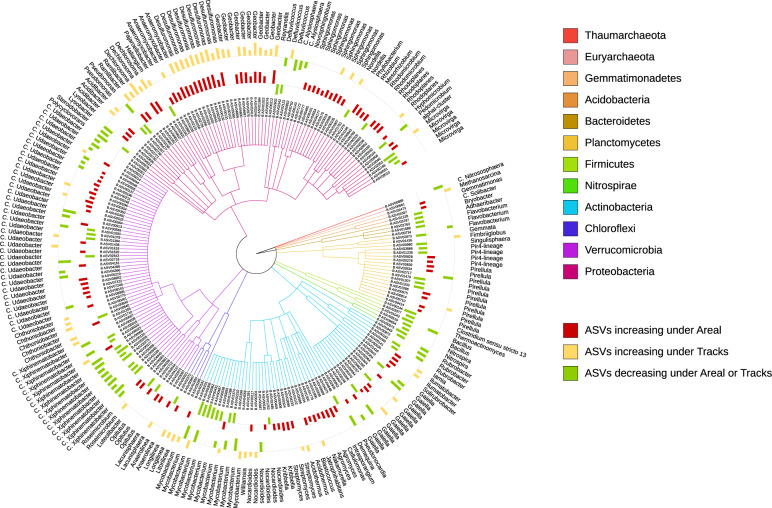
Prokaryotic taxa sensitive to soil compaction. Taxonomic tree showing the bacterial and archaeal ASVs assigned at the genus level and responding significantly to compaction (PERMANOVA, *q* < 0.05). The barplots show the *z*-transformed relative abundances of these ASVs, with the yellow and red bars representing ASVs relatively enriched under the “tracks” and “areal” compaction treatments, respectively, and the green bars representing the ASVs relatively enriched under the “control” treatment.

**Fig. 6:**
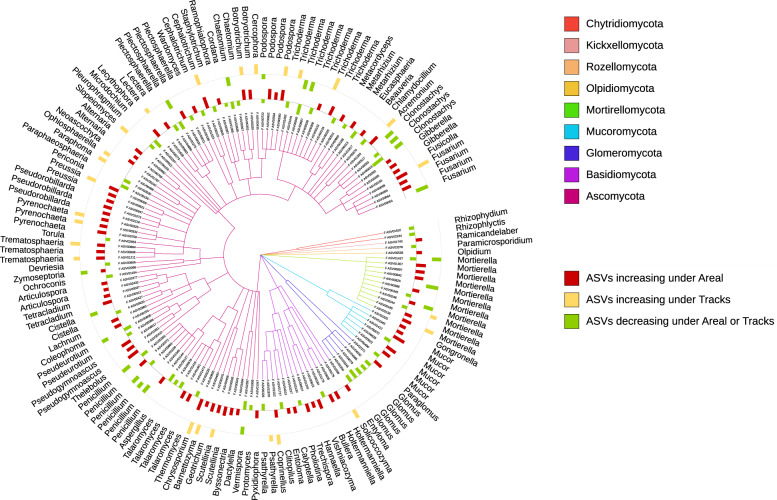
Fungal taxa sensitive to soil compaction. Taxonomic tree showing the fungal ASVs assigned at the genus level and responding significantly to compaction (PERMANOVA, *q* < 0.05). The barplots show the *z*-transformed relative abundances of these ASVs, with the yellow and red bars representing ASVs relatively enriched under the “tracks” and “areal” compaction treatments, respectively, and the green bars representing the ASVs relatively enriched under the “control” treatment.

The sensitive ASVs, assigned at the genus levels, were broadly spread across the taxonomic tree and present in all major phyla (Figs. 5 and 6). Several ASVs with contrasting responses to soil compaction were assigned to the same genus (Figs. 5 and 6). Salient examples of prokaryotic (Fig. 5) and fungal (Fig. 6) genera with ASVs increasing under “tracks” and “areal” compaction (Figs. 5 and 6, yellow and red bars) included *Sphingomonas, Geobacter, Desulfuromonas, Anaeromyxobacter, Dechlorosoma, Lysobacter* (all *Proteobacteria*), *Anaerolinea, Longilinea* (*Chloroflexi*), *Intrasporangium, Cellulomonas, Agromyces, Streptomyces, Micromonospora* (*Actinobacteria*), and *Mathanosarcina* (*Euryarchaeota*), as well as *Mortierella* (*Mortierellomycota*), *Mucor* (*Mucor*) *Tetracladium, Preussia, Podospora, Pseudobillarda, Botryotrichum, Scutellinia, Trematosphaeria*, and *Thelebolus* (all *Ascomycota*). Conversely, genera with ASVs showing higher relative abundance in the uncompacted control plots (Figs. 5 and 6, green bars) included *Candidatus Xiphinematobacter* (*Verrucomicrobia*), *Nitrospira* (*Nitrospirae*), *Mycobacterium, Demequina* (*Actinobacteria*), *Pseudomonas* (*Proteobacteria*), *Bacillus* (*Firmicutes*), *Flavobacterium* (*Bacteroidetes*), and *Candidatus Nitrososphaera* (*Thaumarchaeota*), as well as *Ustilago, Calyptella* (*Basidiomycota*), *Paramicrosporidium* (*Rozellomycota*), *Glomus* (*Glomeromycota*), *Trichoderma, Aspergillus, Penicillium, Devriesia, Periconia, Chlamydocillium, Microdochium, Cordana, Plectosphaerella, Paraphaeosphaeria, Clonostachys*, and *Chaetomium* (*Ascomycota*). In addition to the ASV level response, these shifts in relative abundance were also statistically evaluated by aggregating the data at all assigned taxonomic levels from genus to phylum (Supplementary Data 1). None of the sensitive ASVs presented above revealed a significant time-dependent change in their response to compaction in any agricultural management system (Supplementary Data 1).

### Soil microbial gene abundances

In 2014, *nir*S and *nir*K genes abundances in soil tended to increase with soil compaction under PL and NT when compared to their respective control, whereas they tend to decrease with compaction under CT (Supplementary Fig. 6). In 2017, *nir*S and *nir*K genes abundances in soil did not show any specific trends (Supplementary Fig. 6). However, those changes in *nir*S and *nir*K gene abundances were not statistically significant due to the large variation between blocks. The estimated copy number of the 16S rRNA gene also tended to increase with compaction but was again statistically not supported.

## Discussion

A one-time soil compaction event on an arable field reduced crop yield, altered soil physical properties and changed soil microbial community structure. Many parameters did not fully recover within four growing seasons, although they showed different degrees of resilience. However, the field blocks induced a large variability and sometimes limited the statistical support of the compaction effects. The field site had been used for other field experiments prior to the establishment of the SSO, which might have contributed to the intrinsic heterogeneity across the three blocks and ultimately led to the block-dependent impact of the soil compaction treatment (Fig. 1B). The across-block variability of the compaction effects on plant growth, soil physical properties, and microbial diversity demonstrates the dependency of these compaction effects on the initial edaphic conditions. These environmental variabilities, allowed us, however, to draw more universal conclusions from our data.

### Soil physical properties

Wheeling with a heavy agricultural vehicle increased bulk density in the topsoil (10–15%), and substantially reduced air permeability (60–63%) and gas diffusion (59–66%; Fig. 2). This supports earlier findings that compaction affects large pores, disrupts pore continuity, and strongly decreases fluid transport properties [65]. The decreased gas transport capacity of the soil can ultimately result in poorer aeration and lower levels of oxygen concentration in soil air [66]. In the fourth growing season, bulk density was still higher in the compacted plots of all agricultural management systems without mechanical loosening, i.e., PL and NT (Fig. 2). Soil gas transport properties (air permeability and gas diffusivity) had improved in the compacted plots of all agricultural management systems relative to their non-compacted counterparts within four growing seasons after compaction, yet they tended to remain lower indicating that they may not have fully recovered (see Keller et al. [67]). However, the variability of these soil gas transport properties across the three blocks was substantial, limiting the statistical support of the observations. This can again be explained by the initial variability of the compaction impact (Fig. 1B). Moreover, the variation tends to be higher in the “areal” compaction compared to the “tracks”. This can again be explained by differences in roots and earthworm activities, since both had little other choice than penetrating the soil under the full area compaction, whereas they had the option to avoid the compacted zones in the track only compaction treatments. It has been demonstrated that root systems and earthworms are important actors in the regeneration of compacted soil, but that this is a slow process which needs several years [68, 69].

Overall, the recovery of soil physical properties following compaction is dependent on the severity of compaction, the different soil physical characteristics, such as soil texture, organic carbon content, and clay mineralogy determining shrink-swell processes, climatic conditions that influence dry-rewet and freeze-thaw cycles, vegetation-dependent factors, such as root growth and plant water uptake capacities, soil biological activity, such as earthworm bioturbation as well as mechanical soil management like tillage [5, 70, 71]. Within the three different agricultural management systems, CT facilitated mitigation of compaction effects in the topsoil (Fig. 2). This supports various previous studies, which demonstrated that adapted tillage can improve soil physical and mechanical properties in compacted soils in the short term [72, 73]. However, tillage operations not well adapted to the site conditions can also have detrimental effects by increasing the susceptibility of the topsoil to erosion, facilitate future compaction by destruction of soil aggregates, and ultimately creating a subsoil hardpan layer [72, 73].

### Permanent ley biomass and arable crop yield

Ley biomass and crop yields, especially under NT, were reduced by soil compaction especially in the first two growing seasons (Fig. 3), which can be in agreement with the significant increase in bulk density observed under compaction in PL and NT (Fig. 2). Ley biomass was lower in compacted than non-compacted soils even four growing seasons after compaction, but the large variability across blocks limited statistical support. In contrast, crop yield seemed to have largely recovered from the third growing season onward (Fig. 3). The different compaction effects on plant growth can be explained by different sensitivities of different crops to compaction [9, 17], which varies among crops, but also with the variation of the initial impact among blocks. Moreover, the impact of compaction on crop yield has been reported to be weather dependent, so that longer time series may be needed to quantify long-term trends. Indeed, it has been previously reported that compaction can result in long-term crop yield penalties, but that year-to-year variation can be significant [74].

### Soil microbial diversity

In support of our hypothesis, both soil compaction and agricultural management systems promoted distinct soil microbial communities (Table 1 and Fig. 4). Differences between agricultural management systems were likely attributed to differences in vegetation (PL versus crop rotation with annual crops) and soil management practices (none versus no tillage versus tillage). It is widely accepted that soil microbial communities are strongly shaped by the plants growing in the soil [75], and it has been shown that different tillage practices can influence soil microbial diversity [76]. Microbial differences induced by soil compaction were likely attributed to changes in soil porosity affecting oxygen penetration and diffusion, as well as water availability [25]. However, an increase in microbial richness due to soil compaction as observed in a previous soil compaction study in forests [25], was only confirmed for fungi but not for prokaryotes (Supplementary Figs. 2 and 3). Bacterial abundance approximated by quantification of the 16S rRNA gene did not change with compaction, which is again in contrast with the observation in the previous forest soil compaction study [25]. Interestingly, soil microbial communities showed little structural resilience over the four growing seasons, despite the partial recovery of soil physical properties and crop yields (Table 1). Furthermore, the different soil compaction scenarios, i.e., “tracks” versus “areal”, affected soil microbial communities differently (Fig. 4C, D). There are different potential explanations for these differences. Firstly, “areal” compaction showed a stronger imprint on soil bulk density, air permeability, and gas diffusion than the “tracks” compaction (Fig. 2). Secondly, the uncompacted surrounding soil in the “tracks” compaction treatment might have influenced the microbial communities differently.

However, spatial and temporal effects were the two predominant drivers of soil microbial communities (Table 1 and Supplementary Fig. 4C–F). The spatial differences in microbial community structure across the three blocks already existed before the establishment of the experiment (Supplementary Fig. 4A, B), and can probably be attributed to small differences in soil texture and soil organic carbon content, as well as differences in pH (Fig. 1C) [11]. For example, pH—a strong driver of microbial diversity [77]—differed between blocks A and C by one unit (Fig. 1C). Furthermore, soil texture—another known driver of microbial diversity [78]—showed slightly increasing sand and clay, as well as decreasing silt contents moving from blocks A to B and C (Fig. 1C).

The temporal component was the second strongest driver of prokaryotic and fungal diversity (Table 1 and Supplementary Fig. 4E, F), with a major shift in microbial community structure observed between the second (2015) and the third (2016) growing season. This substantial shift could be attributed to a strong legacy effect of the dry summer of 2015, where precipitation in July and August was 70 and 50% lower compared to the three other growing seasons (source: www.meteoswiss.admin.ch). Precipitation and associated changes in soil moisture are known to have direct short-term, as well as long-term legacy effects on microbial diversity [79].

In contrast to less frequently managed ecosystems like forests [25], this agricultural field has been managed for decades, and thus the microbial communities may have adapted to some degree of soil compaction also in the control plots, resulting in a lower impact of compaction compared to all other factors.

### Compaction-sensitive microbial taxa

Around 10% of the microbial ASVs responded to soil compaction, and these responses were often specific to the agricultural management systems and compaction treatments with very few (28 out of 1237 ASVs) universal responses across all systems and treatments (Figs. 5 and 6, and Supplementary Fig. 5). In the following, the most salient examples and potential ecological mechanisms are discussed in more detail.

The main driver of prokaryotes appeared to be the pore space and oxygen limitation due to the increase in bulk density leading to a reduction in air permeability and gas diffusion [11], as it has been observed in this experiment (Fig. 2 and Supplementary Fig. 6). Bacterial and archaeal species capable of metabolizing under a low partial pressure of oxygen commonly thrive under these conditions [25]. Indeed, bacterial and archaeal genera with known anaerobic lifestyles, such as *Desulfuromonas, Anaeromyxobacter, Geobacter, Anaerolinea, Longilinea, Intrasporangium, Dechlorosoma*, and *Methanosarcina* significantly increased in relative abundance under soil compaction (Fig. 5). Many of these bacterial and archaeal taxa have also been shown to increase in compacted forest soils [25], paddy rice soils [80], or temporarily water-logged agricultural fields [81], and thus might serve as indicators of oxygen-limited soil environments. Analogously, many taxa with aerobic lifestyles can be restricted in compacted soils [21]. In this study, bacterial taxa such as *Demequina, Mycobacterium*, and *Nitrospira*, as well as the archaeal taxa *Candidatus Nitrososphaera*, which are known to exhibit a strictly aerobic lifestyle, significantly decreased in relative abundance in the compacted soils (Fig. 5). *Candidatus Nitrosophaeraceae* and *Nitrospira* are both known as nitrifiers and their reduced relative abundance raises the question of potential interference with important aerobic processes, such as nitrification in compacted soils, as already suggested previously [82]. Reduced nitrate availability as a consequence of limited nitrification, as well as enhanced denitrification under soil compaction [19] can ultimately impact plant growth and crop yield [21, 83], which can also partially explain the initial decrease in plant yield observed in the PL and the crop rotations.

In order to further support this hypothesis, *nir*S and *nir*K genes abundances were assessed. Soil compaction tended to increase *nir*S and *nir*K gene abundances in 2014 under PL and NT, but not under CT (Supplementary Fig. 6). It is well documented that soil compaction can increase soil potential denitrification [84, 85] due to the decrease in soil aeration. Our observations confirm the limitation in oxygen infiltration and gas diffusion observed in the field with compaction under PL and NT at the beginning of the experiment and the mitigation of oxygen depletion under CT (Fig. 2). However, those increases in gene abundances were not statistically supported, which again can be explained by the initial variability of the compaction impact (Fig. 1B), and an apparent lack of differences in *nir*S and *nir*K gene abundances between compacted and uncompacted plots in block A.

Aside from soil pore space and oxygen limitation, plants are another main driver of prokaryotic and fungal communities. In our study, many known potentially beneficial and/or pathogenic plant-associated bacteria, such as *Mycobacterium, Flavobacterium, Bacillus*, and *Pseudomonas* [86] decreased under soil compaction (Figs. 5 and 6). Moreover, several plant-associated fungi also decreased under soil compaction, which was the case for the arbuscular mycorrhizal fungus *Glomus* [87], the plant-symbiotic biocontrol agent *Trichoderma* [88], or the plant growth promoting fungi *Aspergillus* and *Penicillium* [89]. Many of the fungal genera decreasing under soil compaction included potential phytopathogens, such as *Devriesia, Clonostachys, Plectosphaerella, Paraphaeosphaeria, Periconia, Chlamydocillium, Microdochium, Ustilago, Calyptella*, and *Cordana*. A reduced development of the plant root system [16, 90] and an increase in plant mortality [91] due to soil compaction are directly related to the reduced plant yield observed in this study (Fig. 3), and may also explain the decline in plant-associated bacteria and fungi.

An increase in plant mortality or root die-off can lead to an accumulation of decaying plant tissue in compacted soils, which in turn can explain the increase in some potentially cellulolytic bacteria, such as *Lysobacter, Cellulomonas, Agromyces, Sphingomonas, Streptomyces*, and *Micromonospora* [92–94]. Similarly, the majority of saprophytic fungal genera that significantly increased under soil compaction, such as *Pseudorobillarda, Botryotrichum, Preussia, Scutellinia, Trematosphaeria, Mortierella, Mucor*, *Tetracladium*, and *Thelebolus* are known to be involved in lignocellulose degradation [63]. We further observed that the bacterial genus *Candidatus Xiphinematobacter* and the fungal genus *Paramicrosporidium* decreased under compaction. Those microorganisms are known to be associated with nematodes [95] and amoeba [96], respectively, organisms which may have been limited in their movements in our study due to the reduction in porosity and water flow in the compacted soils [97].

Overall, our results are in line with our hypothesis and consistent with previous observations in compacted forest soils [25], i.e., that soil compaction favors the abundance of anaerobic and saprotrophic microorganisms, whereas aerobic and host-associated organisms are more negatively affected. However, this list of potential explanations is, of course, not exhaustive since soil compaction and its decrease in average pore size can also provide protection for bacteria and archaea against predation by protozoa [98] and limit hyphal growth of fungi [18]. We also recognize that our ability to draw conclusions about possible lifestyles of bacteria, archaea, and fungi from taxonomic identity and information from the available literature alone is limited, and may be missing some other relevant associations. Interestingly, none of the genera responding to soil compaction did recover over the four growing seasons, which corroborated the lack of complete resilience of the microbial community structure, which agrees with the persistent differences in bulk densities.

## Conclusion

The extent to which a single soil compaction event has altered the soil microbial system over four growing seasons and the lack of complete resilience despite ongoing soil management practices, such as tillage and crop production activities is remarkable. A relative increase of anaerobically metabolizing prokaryotes and saprotrophic fungi under soil compaction compared to the uncompacted control accompanied by generally negative effects on microorganisms with aerobic or plant-host-associated lifestyles appears to be a unifying concept that agrees with previous studies carried out in forest soils. Given the small but consistent and long-lasting effect of this single disturbance event, the findings raise concerns about potential cumulative effects of soil compaction events associated with regular agricultural operations and repeated compaction events on the same field. The legacy effects of soil compaction on the microbiome and its functions, as well as the consequences for soil productivity deserve further attention in the debate of how to make cropping systems more sustainable.

## Supplementary information

Supplementary Table S1

Supplementary information
