# Supplementary material for: Limited resilience of the soil microbiome to mechanical compaction within four growing seasons of agricultural management
Source: ISME Commun. 2021 Aug 31;1:44. doi: 10.1038/s43705-021-00046-8 (PMC9723577; doi:10.1038/s43705-021-00046-8)
Supplement: Supplementary file 1 — Supplementary Table S1 [file 43705_2021_46_MOESM1_ESM.docx]

Table S1: Soil compaction and agricultural management effects on soil microbial α diversity with Observed Richness (S), Pielou’s Evenness (J) and Shannon Diversity (H)

| Observed Richness (S) | Bacteria | | | |  |  | Fungi | | | |
| --- | --- | --- | --- | --- | --- | --- | --- | --- | --- | --- |
|  | PERMANOVA^1^ | |  | PERMDISP^2^ |  |  | PERMANOVA1 | |  | PERMDISP^2^ |
|  | F (P) | R^2^ |  | F (P) |  |  | F (P) | R^2^ |  | F (P) |
| Treatment | 5.20 (0.010) | 0.01 |  | 0.72 (0.486) |  |  | 13.69 (0.001) | 0.05 |  | 2.18 (0.102) |
| Management | 6.23 (0.005) | 0.01 |  | 1.99 (0.127) |  |  | 71.21 (0.001) | 0.25 |  | 0.08 (0.933) |
| Date | 246.29 (0.001) | 0.52 |  | 2.65 (0.056) |  |  | 21.71 (0.001) | 0.11 |  | 4.04 (0.010) |
| Block | 154.59 (0.001) | 0.22 |  | 4.19 (0.016) |  |  | 5.55 (0.001) | 0.12 |  | 2.77 (0.072) |
| Treatment × Management | 5.47 (0.001) | 0.02 |  | 1.11 (0.349) |  |  | 0.60 (0.673) | <0.01 |  | 1.54 (0.155) |
| Treatment × Date | 0.43 (0.862) | <0.01 |  | 1.93 (0.040) |  |  | 1.67 (0.123) | 0.02 |  | 1.67 (0.077) |
| Management × Date | 1.17 (0.325) | 0.01 |  | 1.51 (0.129) |  |  | 4.35 (0.002) | 0.05 |  | 0.85 (0.574) |
| Treatment × Management × Date | 1.32 (0.213) | 0.01 |  | 1.41 (0.058) |  |  | 0.44 (0.945) | 0.01 |  | 1.07 (0.343) |
| Residual |  | 0.20 |  |  |  |  |  | 0.49 |  |  |

| Pielou’s Evenness (J) | Bacteria | | | |  |  | Fungi | | | |
| --- | --- | --- | --- | --- | --- | --- | --- | --- | --- | --- |
|  | PERMANOVA^1^ | |  | PERMDISP^2^ |  |  | PERMANOVA1 | |  | PERMDISP^2^ |
|  | F (P) | R^2^ |  | F (P) |  |  | F (P) | R^2^ |  | F (P) |
| Treatment | 10.12 (0.001) | 0.03 |  | 0.72 (0.507) |  |  | 1.02 (0.365) | 0.01 |  | 0.16 (0.877) |
| Management | 3.66 (0.019) | 0.01 |  | 1.99 (0.146) |  |  | 5.72 (0.004) | 0.03 |  | 0.20 (0.849) |
| Date | 38.77 (0.001) | 0.13 |  | 2.65 (0.050) |  |  | 2.22 (0.075) | 0.02 |  | 0.61 (0.649) |
| Block | 225.31 (0.001) | 0.49 |  | 4.20 (0.011) |  |  | 1.54 (0.217) | 0.01 |  | 0.50 (0.655) |
| Treatment × Management | 4.81 (0.004) | 0.02 |  | 1.11 (0.358) |  |  | 0.32 (0.885) | <0.01 |  | 0.76 (0.685) |
| Treatment × Date | 0.34 (0.929) | <0.01 |  | 1.93 (0.034) |  |  | 0.98 (0.483) | 0.02 |  | 0.64 (0.841) |
| Management × Date | 2.02 (0.073) | 0.01 |  | 1.51 (0.107) |  |  | 1.42 (0.182) | 0.03 |  | 0.93 (0.501) |
| Treatment × Management × Date | 0.69 (0.750) | 0.01 |  | 1.41 (0.070) |  |  | 0.58 (0.863) | 0.02 |  | 0.75 (0.874) |
| Residual |  | 0.31 |  |  |  |  |  | 0.86 |  |  |

| Shannon Diversity (H) | Bacteria | | | |  |  | Fungi | | | |
| --- | --- | --- | --- | --- | --- | --- | --- | --- | --- | --- |
|  | PERMANOVA^1^ | |  | PERMDISP^2^ |  |  | PERMANOVA1 | |  | PERMDISP^2^ |
|  | F (P) | R^2^ |  | F (P) |  |  | F (P) | R^2^ |  | F (P) |
| Treatment | 8.81 (0.001) | 0.02 |  | 3.63 (0.026) |  |  | 2.19 (0.094) | 0.01 |  | 0.29 (0.765) |
| Management | 0.20 (0.829) | 0.04 |  | 4.80 (0.011) |  |  | 12.31 (0.001) | 0.07 |  | 0.11 (0.906) |
| Date | 80.84 (0.001) | 0.23 |  | 3.29 (0.017) |  |  | 2.34 (0.073) | 0.02 |  | 0.95 (0.418) |
| Block | 218.34 (0.001) | 0.43 |  | 28.42 (0.001) |  |  | 2.10 (0.118) | 0.01 |  | 0.09 (0.927) |
| Treatment × Management | 5.18 (0.001) | 0.02 |  | 2.75 (0.006) |  |  | 0.32 (0.876) | <0.01 |  | 0.83 (0.621) |
| Treatment × Date | 0.26 (0.957) | <0.01 |  | 2.09 (0.026) |  |  | 1.08 (0.373) | 0.02 |  | 0.79 (0.708) |
| Management × Date | 1.34 (0.224) | 0.01 |  | 1.74 (0.056) |  |  | 1.38 (0.222) | 0.02 |  | 0.89 (0.540) |
| Treatment × Management × Date | 0.88 (0.553) | 0.01 |  | 1.23 (0.181) |  |  | 0.53 (0.913) | 0.02 |  | 0.78 (0.860) |
| Residual |  | 0.28 |  |  |  |  |  | 0.82 |  |  |

^1^ Effects of main factors and their interactions as assessed by univariate permutational analysis of variance (PERMANOVA). Values indicate the F-ratio (F), the level of significance (P) and the explained variance (R^2^).

^2^ Heterogeneity of variance assessed by permutational analysis of univariate dispersion (PERMDISP). Values indicate the F-ratio (F) and the level of significance (P)
