# Supplementary material for: Limited resilience of the soil microbiome to mechanical compaction within four growing seasons of agricultural management
Source: ISME Commun. 2021 Aug 31;1:44. doi: 10.1038/s43705-021-00046-8 (PMC9723577; doi:10.1038/s43705-021-00046-8)
Supplement: Supplementary file 2 — Supplementary information [file 43705_2021_46_MOESM2_ESM.pdf]

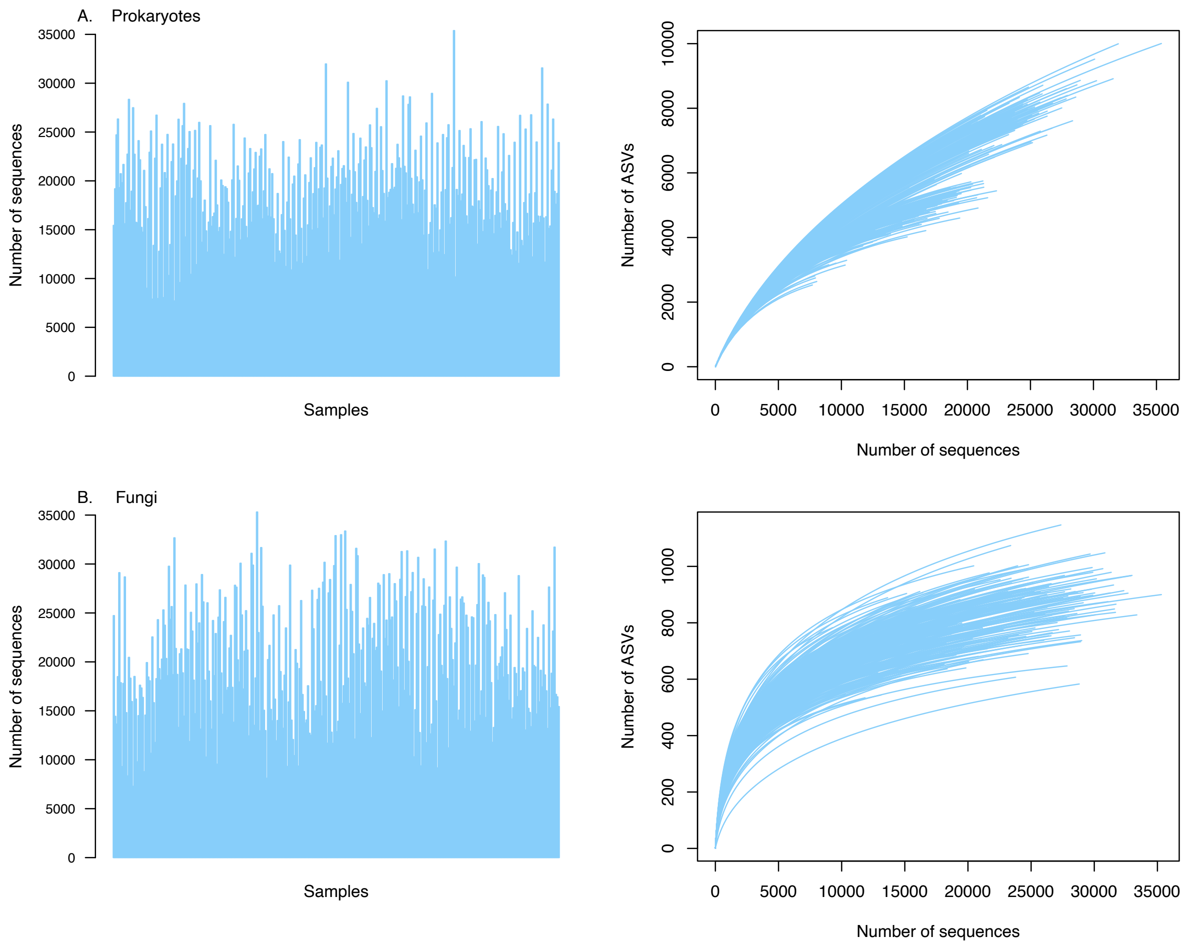

Supplementary Figure 1 : Total number of sequences per sample and their associated rarefaction curves for bacteria/archaea (A) and Fungi (B)

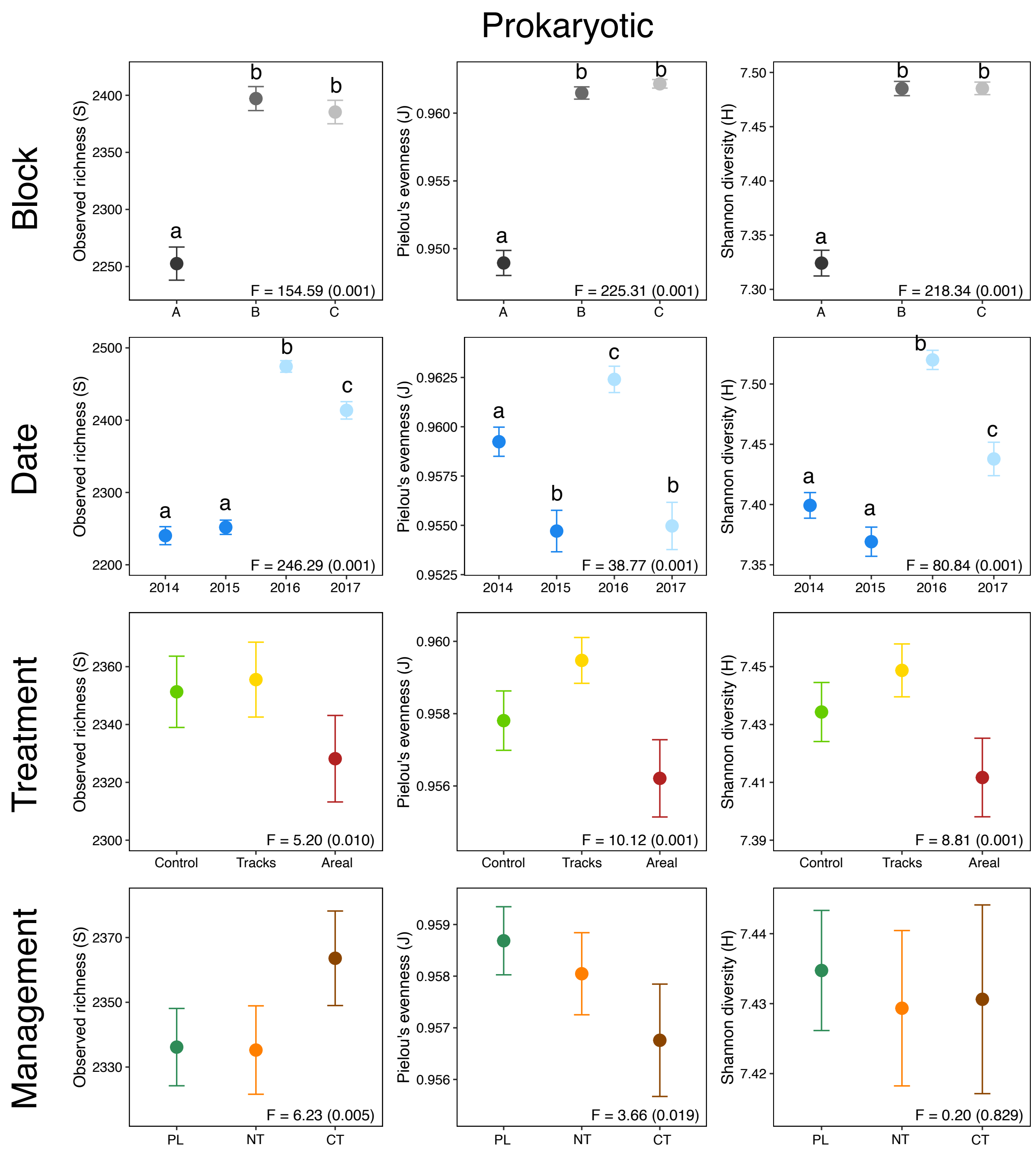

Supplementary Figure 2 : Differences in soil bacteria and archaea observed richness, evenness and Shannon index across the three blocks (A,B and C); across the four sampling time points (2014, 2015, 2016 and 2017), across the three compaction treatment (Control, Tracks and Areal) and across the three agricultural management systems (permanent ley (PL), crop rotation without tillage (NT) and crop rotation with tillage (CT)). The data represents the mean (±se) from each condition with n=108. The values at the bottom right corner indicate the F-ratio (F) with it associated p-value in brackets obtained by univariate permutational analysis of variance (PERMANOVA). The letters indicate significant differences obtained by the post-hoc-test pairwise.perm.manova function.

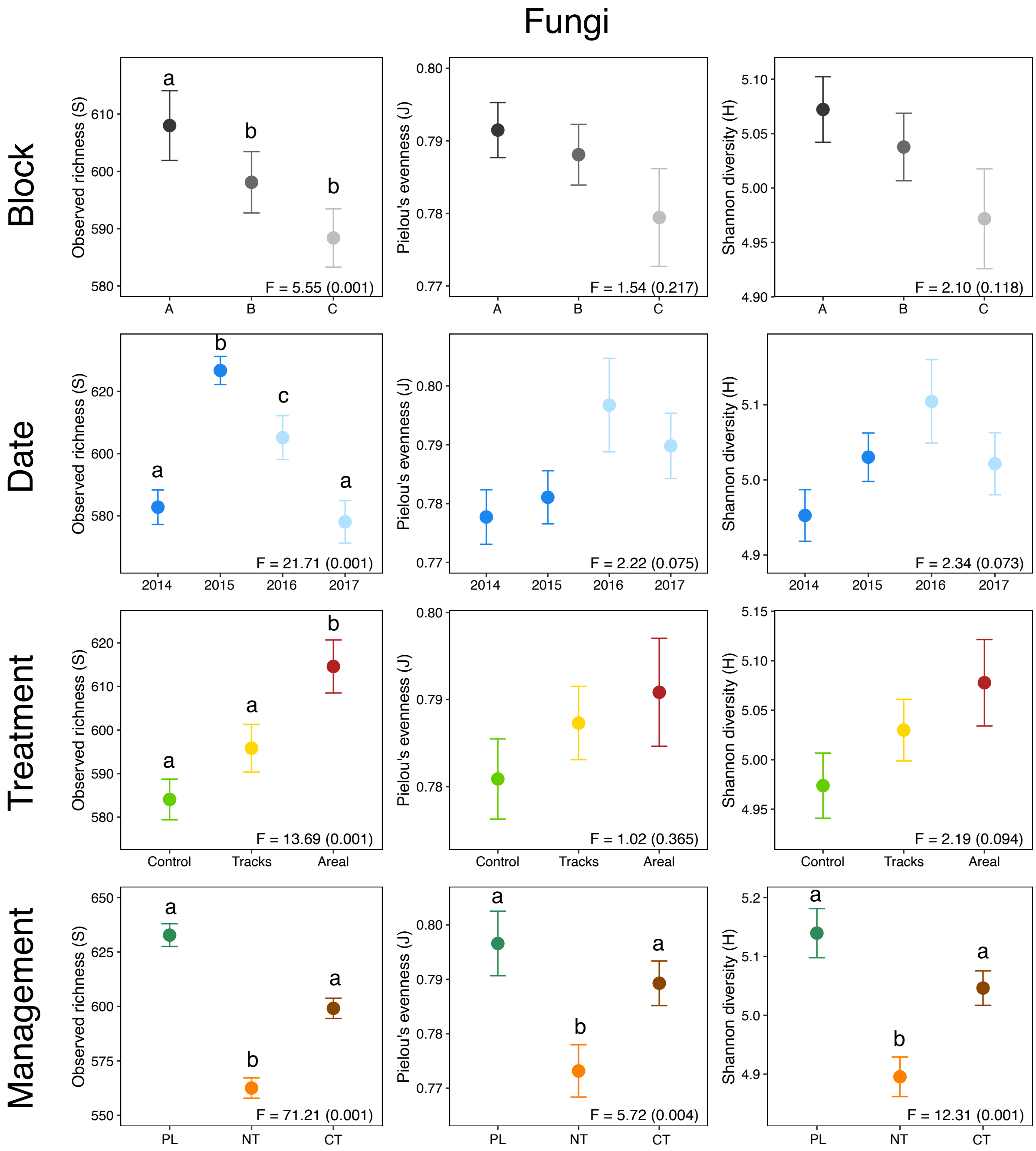

Supplementary Figure 3 : Differences in soil fungi observed richness, evenness and Shannon index across the three blocks (A,B and C); across the four sampling time points (2014, 2015, 2016 and 2017), across the three compaction treatment (Control, Tracks and Areal) and across the three agricultural management systems (permanent ley (PL), crop rotation without tillage (NT) and crop rotation with tillage (CT)). The data represents the mean (±se) from each condition with n=108. The values at the bottom right corner indicate the F-ratio (F) with it associated p-value in brackets obtained by univariate permutational analysis of variance (PERMANOVA). The letters indicate significant differences obtained by the post-hoc-test pairwise.perm.manova function.

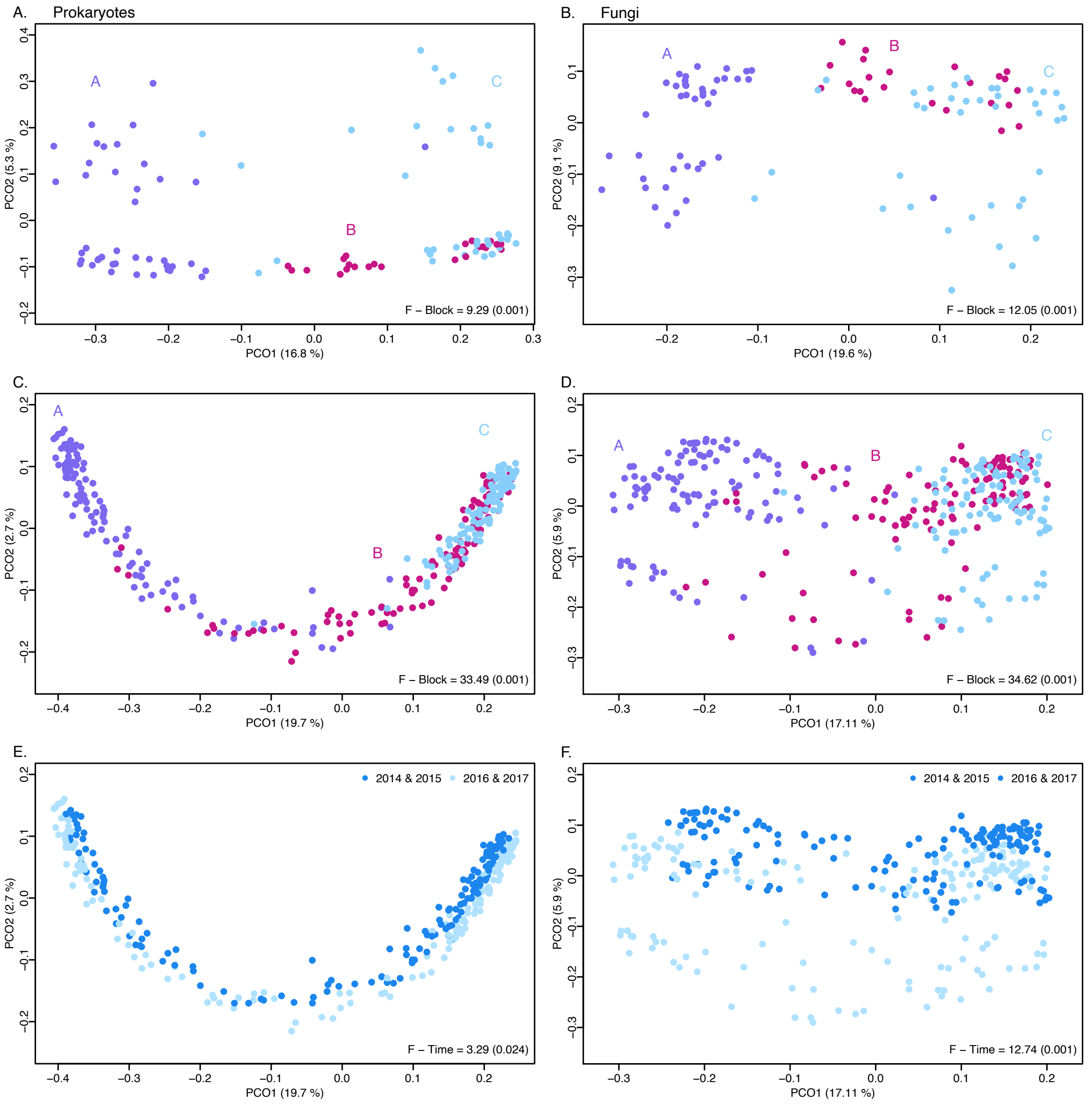

Supplementary Figure 4 : Unconstrained ordination (PCoA) before soil compaction (2013-2014) for bacteria (A) and fungi (B) and after soil compaction (2014-2017) for bacteria (C and E) and for fungi (D and F) colored coded by block (A, B, C, D) or time (E, F). PERMANOVA F and P values of the factors are provided in the plot corners.

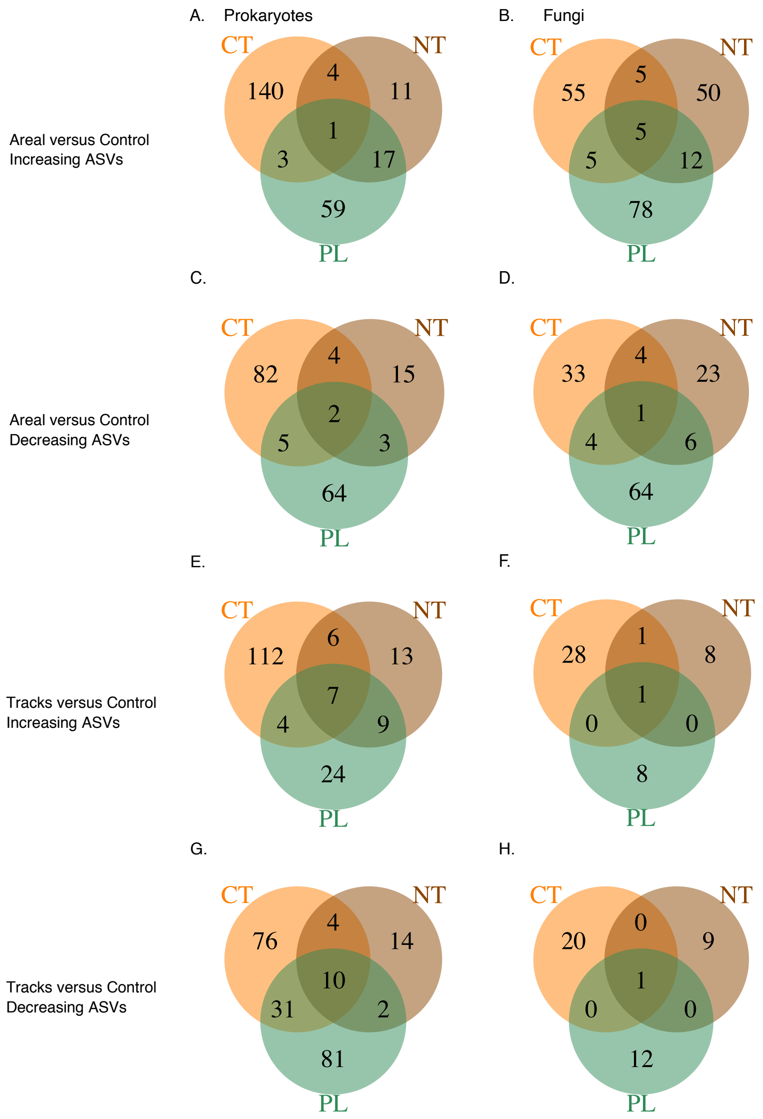

Supplementary Figure 5 : Venn diagram of the statistically significant compaction sensitive bacterial (A, C, E, G) and fungal ASVs (B, D, F, H) for the three agricultural management systems for areal (A-D) and track compaction (E-H). ASVs increasing under compactions are representing in A and E for bacteria and B and F for fungi whereas ASVs decreasing under compaction are representing in C and G for bacteria and D and H for fungi. PL, permanent ley; NT, no-tillage crop rotation; CT, conventional tillage crop rotation.

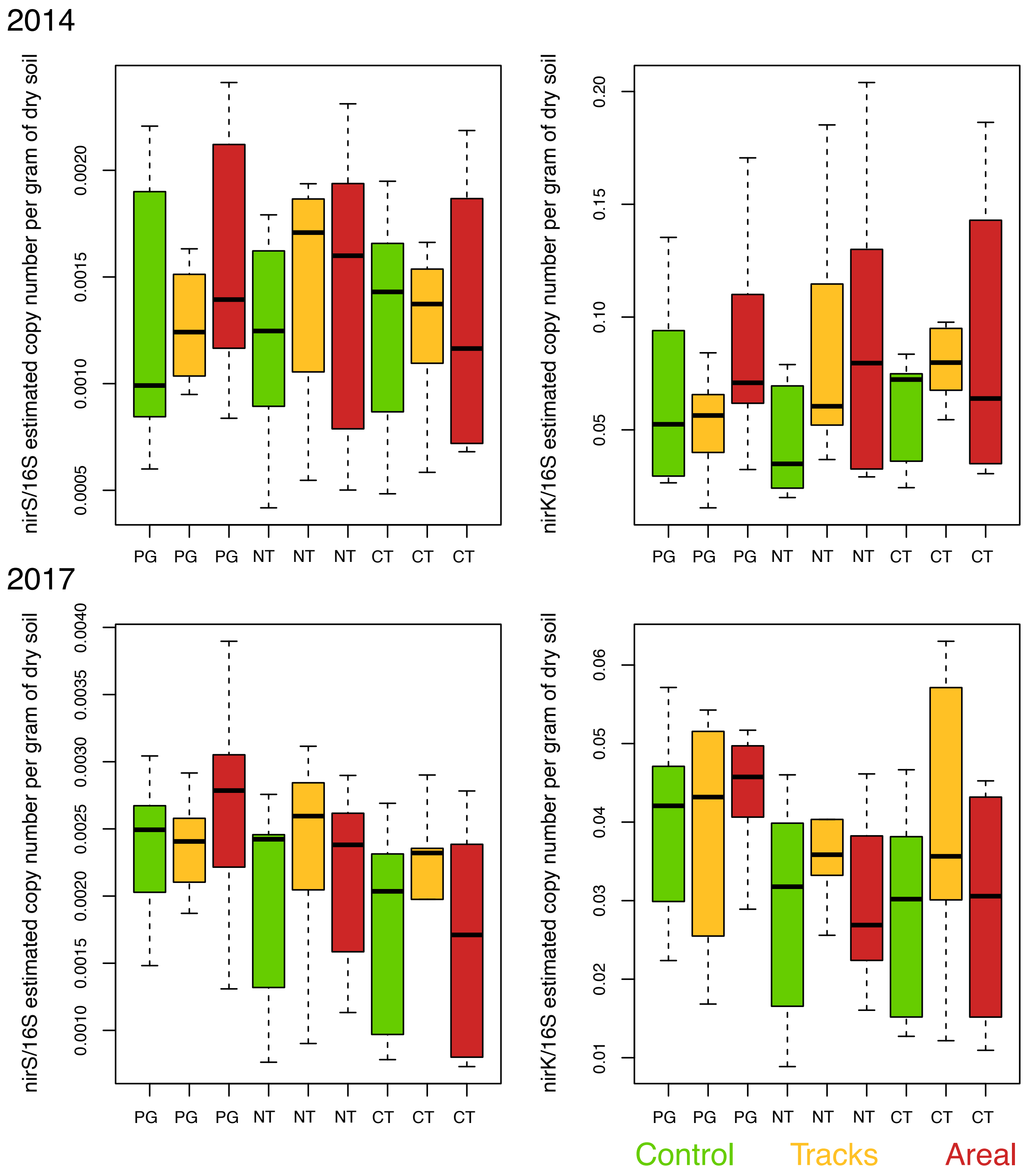

Supplementary Figure 6 : nirS and nirK copy number per gram of dry soil normalized by the 16S gene in the Control, Tracks and Areal compaction treatments for the permanent ley (PL) as well as the crop rotations with conventional tillage (CT) and no tillage (NT) in 2014 and 2017.
